# Frail is not fail: Limited impact of comorbidities on non‐relapse mortality and safety in patients with LBCL treated with CAR‐T

**DOI:** 10.1111/bjh.20222

**Published:** 2025-06-23

**Authors:** Eugenio Galli, Roberta Di Blasi, Ilaria Pansini, Caterina Cristinelli, Come Bommier, Ilenia De Bernardis, Alessandro Corrente, Marcello Viscovo, Luca Montini, Patrizia Chiusolo, Stefan Hohaus, Federica Sorà, Catherine Thieblemont, Simona Sica

**Affiliations:** ^1^ Department of Laboratory and Hematological Sciences Fondazione Policlinico Universitario A. Gemelli IRCCS Rome Italy; ^2^ Hemato‐Oncology Department Assistance Publique–Hôpitaux de Paris (AP‐HP), Saint‐Louis Hospital Paris France; ^3^ Hematology Section, Department of Radiological and Hematological Sciences Catholic University of the Sacred Heart Rome Italy; ^4^ Department of Anesthesia, Intensive Care, and Clinical Toxicology Fondazione Policlinico Universitario A. Gemelli IRCCS Rome Italy; ^5^ Université Paris Cité Paris France; ^6^ Inserm U1153, Saint‐Louis Hospital Paris France

**Keywords:** CAR‐T, cell therapy, non‐Hodgkin‐S lymphoma, tolerability

## Abstract

We applied three major comorbidity scoring systems—CIRS, HCT‐CI, and Severe4—to a cohort of 379 patients with LBCL treated with CAR‐T therapy. A high comorbidity burden was identified in 7% to 34% of patients, depending on the score used. However, a high comorbidity burden did not negatively impact the tolerability of CAR‐T treatment, including the incidence of CRS, or hematologic toxicity. The use of tocilizumab and corticosteroids was comparable between patients with low and high comorbidity burden, as was the cumulative incidence of non‐relapse mortality.

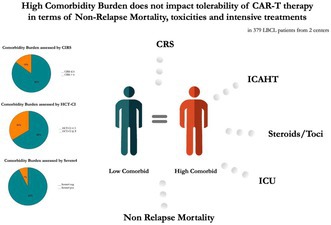

To the Editor,

Non‐relapse mortality (NRM) following treatment with chimeric antigen receptor T cells (CAR‐T) can range between 5.7% and 10.6%.^1^ The main causes of NRM are primarily infections, cytokine release syndrome (CRS) or immune effector cell‐associated neurotoxicity syndrome (ICANS) (10%–14%), cardiovascular events (7%–12%) and secondary neoplasms (6%–8%), with myelodysplastic syndromes (MDS) or acute myeloid leukemia (AML) representing approximately 33% of these cases.^1^, ^2^ Existing laboratory‐based risk scores, like the CAR‐HEMATOTOX, the modified endothelial activation and stress index (mEASIX) and the clonal haematopoiesis risk score (CHRS), mainly address acute and late toxicities, like CRS, ICANS, infections and immune effector cell‐associated haematotoxicity (ICAHT) or secondary myeloid neoplasms.^3^, ^4^, ^5^, ^6^, ^7^

Comorbidities have received less attention compared to biological markers, likely due to the strict regulation of CAR‐T treatment access in both clinical trials and real‐world settings, particularly in terms of candidate ‘fitness’. In many countries, physicians must confirm a good general performance status and organ function to approve CAR‐T treatment,^8^ and frailty may remain a barrier to CAR‐T access in many cases. The appropriate assessment tool for comorbidities is still a matter of debate. The cumulative illness rating scale (CIRS) determines the cumulative burden of comorbidities^9^ from a geriatric point of view. Recently, a multicentre study found that a CIRS score of 7 or higher was associated with reduced overall survival (OS) in patients treated with CAR‐T.^10^ The same group identified that a CIRS score ≥3 in the respiratory, upper gastrointestinal, hepatic or renal systems—referred to as ‘Severe4’—predicted both progression‐free survival (PFS) and OS.^11^ The haematopoietic cell transplantation‐specific comorbidity index (HCT‐CI) evaluates 14 organ systems focusing on the risk of post‐transplant complications and mortality and may predict OS after CAR‐T.^12^ Lymphoma‐oriented (CIRS and Severe4) and cell therapy‐oriented (HCT‐CI) scores are detailed in Supporting Information S1. However, available scores primarily address the impact of comorbidities on PFS and OS, and no studies have specifically investigated their role in NRM after CAR‐T.

We aimed to explore how the baseline comorbidity burden impacts safety when treating patients with CD19‐directed CAR‐T cells, evaluating the CIRS, Severe4 and HCT‐CI towards the NRM, CRS and ICANS assessed and treated according to the current European consensus.^13^, ^14^ A positive Severe4, a CIRS score greater than 6 and/or an HCT‐CI score of 3 or more were used to identify ‘frailty’. Additionally, we sought if frail patients received more likely tocilizumab, steroids or admission to intensive care unit (ICU).

We studied 379 consecutive patients treated with CD19‐directed CAR‐T cells for large B‐cell lymphomas between October 2018 and October 2024 at two major European hub centres (Hôpital Saint Louis, APHP, Paris, France, and Fondazione Policlinico Universitario A. Gemelli IRCCS, Rome, Italy). Characteristics of the study population are summarized in Table 1. Statistical analysis and adherence to STROBE guidelines are reported in Supporting Information (S2 and S3). All participants provided informed consent for the anonymized use of their data. The study was conducted in accordance with the Helsinki Declaration and was approved by the local ethics committee (ID 4879 Prot 0020777/22 Amendment 09/2024). The median follow‐up was 12 months. PFS was 51% and 46% at 1 and 5 years, respectively, while NRM was 4% and 8% at 1 and 5 years respectively. No significant differences were observed between the treating centres in PFS or NRM (*p* = 0.885 and *p* = 0.340 respectively).

**TABLE 1:** Characteristics of the study population. Variables are detailed for the overall study population and detailed for highly comorbid patients, identified with CIRS >6, Severe4 or HCT ≥3. Male patients show higher comorbidity burden, and elderly patients tend to show higher comorbidities when assessed with CIRS.

		Total population	CIRS >6	Severe4+	HCT‐CI ≥3
Patients *n* (% or range)		379 (100)	56 (14.8)	28 (7.4)	128 (33.8)
Gender	Males	228 (60.1)	42 (75)*	25 (89)*	99 (77)*
Age	Median (range)	62 (21–86)	66.5 (40–79)*	67 (49–79)*	63 (23–86)*
	>70 years	94 (24.8)	22 (39)*	8 (29)	34 (26)
	>75 years	29 (7.6)	8 (14)*	4 (14)	13 (10)
Diagnosis	DLBCL	292 (77)	41 (73)	21 (75)	100 (78)
	PMBL	17 (4.5)	1 (2)	0 (0)	3 (2)
	tFL	70 (18.5)	14 (25)	7 (25)	25 (20)
Previous lines	Median	2 (1–8)	2 (1–7)	2 (1–7)	2 (1–8)
Type of CAR‐T	Axi‐cel	217 (57.2)	29 (52)	17 (61)	69 (54)
	Liso‐cel	15 (4)	2 (4)	0 (0)	4 (3)
	Tisa‐cel	47 (38.8)	25 (44)	11 (39)	55 (43)
Status before CAR‐T	CR‐PR	106 (28.8)	19 (34)	9 (32)	31 (25)
	SD‐PD	262 (71.2)	37 (66)	19 (68)	93 (75)
ECOG	0–1	328 (86.5)	43 (77)	24 (86)	109 (87)
	2–4	51 (13.5)	13 (23)	4 (14)	17 (13)
LDH	Elevated	194 (53.1)	26 (46)	14 (52)	59 (49)
Stage	III–IV	276 (76.2)	44 (83)	23 (82)	96 (79)
CAR‐HEMATOTOX	Low	175 (47.7)	23 (41)	9 (33)	60 (48)
	Intermediate‐High	192 (52.3)	33 (59)	18 (67)	64 (52)
mEASIX	Median (range)	3.97 (−1.79–11.94)	4.23 (−0.7–11.4)	4.1 (1.11–11.9)	3.85 (0.96–11.9)
	>6.8	52 (17)	6 (13)	4 (16)	15 (14)

Abbreviations: CAR‐T, chimeric antigen receptor T; CIRS, cumulative illness rating scale; CR, complete response; DLBCL, diffuse large B‐cell lymphoma; ECOG, Eastern Cooperative Oncology Group; HCT‐CI, hematopoietic cell transplantation‐comorbidity index; mEASIX, modified endothelial activation and stress index; PD, progressive disease; PMBL, primary mediastinal B‐cell lymphoma; PR, partial response; SD, stable disease; tFL, transformed follicular lymphoma.

* Statistical difference with *p* value <0.05.

Despite the population is somehow selected to be fit enough to receive CAR‐T (in Italy, a formal checklist is required from the national drug agency AIFA), we found that a considerable proportion (7.4%–33.8%) could be considered ‘frail’ according to comorbidity scoring systems. The cumulative CIRS score was similar across centres (CIRS >6 14.3% vs. 16.3% in French and Italian cohort, respectively, *p* = 0.669). CIRS ranged from 0 (12.4% of patients) to 13 (0.8% of patients), with a median of 3. A CIRS higher than 6 was found in 56 patients (14.8%). Among the systems, the respiratory tract had the highest cumulative burden of comorbidity, mainly due to long‐term smoking exposure. Systems with the higher comorbidity burden were vascular, respiratory and urogenital systems, largely due to previous thromboembolic events, severe smoking exposure and urogenital cancers (Supporting Information S1). The Severe4 score was positive in 28 patients (7.4%). The distribution of the HCT‐CI score (range: 0–10, median: 2) was similar across centres (HCT‐CI ≥3 in 34.8% vs. 30.2% in French and Italian cohort, respectively, *p* = 0.773), with 128 patients (33.8%) having three or more points overall. Notably, CIRS and HCT‐CI were conceived for different purposes, being the former a geriatric derived tool later applied to oncology, and the latter emphasizing key areas for transplantation, such as the cardiovascular system, obesity, liver and lungs. Although CIRS and HCT‐CI were concordant in identifying the comorbidity burden in most cases, 24.8% of patients were identified as ‘highly comorbid’ (CIRS >6 or HCT‐CI ≥3) by only one of the two scores. The complete system‐by‐system report of comorbidity distribution is detailed in the Supporting Information S1.

Cytokine release syndrome occurred in 85.4% of patients, with grades 1, 2, 3 and 4 observed in 46.8%, 31.5%, 6.6% and 0.5% of patients respectively. The median CIRS score was 3 for both patients who developed CRS graded 2–4 and those with no CRS or grade‐1 CRS (*p* = 0.703). The risk of developing CRS graded 2–4 in patients with a CIRS greater than 3 was not increased (odds ratio [OR]: 0.63, 95% CI: 0.36–1.09, *p* = 0.101). Similarly, neither Severe4+ patients (OR: 0.63, 95% CI: 0.28–1.40, *p* = 0.262) nor patients with an HCT‐CI score of 3 or more (OR: 0.89, 95% CI: 0.66–1.19, *p* = 0.446) had a higher risk of developing severe CRS. In multivariate analysis, CRS was independently predicted by higher Eastern Cooperative Oncology Group (ECOG) and mEASIX scores, while patients treated with liso‐cel had a lower risk (S4). ICANS developed in 35% of patients, primarily of grade 1 (16.6%) and grade 2 (9.1%), with one case of grade 5. The median CIRS score was 3 for both groups. In univariate analysis, a CIRS greater than 6 did not increase the risk of developing grade 2–5 ICANS (OR: 1.47, 95% CI: 0.85–2.54, *p* = 0.163) nor did an HCT‐CI of 3 or more (OR: 0.98, 95% CI: 0.68–1.42, *p* = 0.944) or a positive Severe4 score (OR: 0.73, 95% CI: 0.26–2.05, *p* = 0.559). After adjustment in multivariable analysis, we observed a possible correlation between increased neurological toxicities and higher comorbidity burden, as indicated by two out of three comorbidity scores (S4). ICANS occurred less frequently in patients treated with liso‐cel or tisa‐cel compared to axi‐cel, as well as in those with tFL compared to DLBCL (S4). Early and late ICAHT occurred in 88.5% and 69.4% of patients, respectively, with 47.2% and 45.7% of these cases being grade 2 or higher. Once again, a higher comorbidity burden, as assessed by CIRS, Severe4 and HCT‐CI, did not predict the onset of early or late ICAHT grade 2–4 (Supporting Information S4). ICAHT was strongly predicted by CAR‐HEMATOTOX, with lower incidence observed in patients treated with tisa‐cel (and liso‐cel for early ICAHT). Among all patients, tocilizumab and steroids were administered in 33% and 27% of cases respectively. Boluses of prednisone were administered in 6% of patients. Overall, 26% of patients required hospitalization in ICU. Patients with a higher burden of comorbidities did not differ from others in terms of receiving tocilizumab, steroids or ICU admission (data not shown).

The cumulative incidence of NRM was not significantly different between patients with a CIRS up to 6 versus greater than 6 (*p* = 0.877) nor between patients with an HCT‐CI of 0–2 versus 3 or more (*p* = 0.828), or when dichotomized by Severe4 (*p* = 0.535). This was confirmed also when restricting to elderly patients aged 70 years or more (Supporting Information S5), although the sample size in this subgroup was limited. In this context, the concept of ‘frailty’ extends beyond comorbidities to include factors such as functional autonomy (assessed by ADL/IADL scales), psycho‐cognitive status and social support (e.g. the presence of a caregiver).

Median OS was 65% and 47% at 1 and 5 years, respectively, with no difference according to high burden of CIRS (*p* = 0.156), Severe4 (*p* = 0.356) or HCT‐CI (*p* = 0.532). Median PFS was 51% and 46% at 1 and 5 years, respectively, again with no difference according to the comorbidity burden (data not shown).

The main causes of NRM were infections (52%), CRS or ICANS (17%) and miscellaneous causes (including 3 cases of therapy‐related acute myeloid leukaemia, cardiac events and haemophagocytosis), occurring at a median of 0.5 months, 16 months and 18.5 months after CAR‐T infusion respectively. We then analysed the predictors of NRM: clinically relevant variables were selected based on prior literature and their potential association with outcomes after CAR‐T therapy. The complete analysis is detailed in Table 2. Overall, no patient‐related factors, including age, ECOG performance status or comorbidity burden, significantly impacted the risk of NRM (Figure 1), while we found that patients with elevated mEASIX and developing early ICAHT were at higher risk.

**TABLE 2:** Analysis on the predictive potential towards non‐relapse mortality of main variables (Cox Regression). The variables included in the univariate analysis were preselected based on clinical relevance, prior literature and consistency with the study's primary end‐points. Specifically, we considered demographic factors (age, gender), disease‐related characteristics (disease stage, LDH levels, disease status at the time of CAR‐T infusion), treatment‐related variables (type of CAR‐T product, previous autologous stem cell transplantation [ASCT]), performance and comorbidity scores (ECOG, CIRS, HCT‐CI, Severe4), as well as toxicity‐related variables (severity of CRS and ICANS, and the occurrence of early or late ICAHT). This selection was intended to reflect factors known or hypothesized to influence outcomes after CAR‐T therapy, while avoiding an overly data‐driven approach. This clinical filtering was applied to avoid an indiscriminate data‐driven approach and ensure that only variables with plausible biological or clinical rationale were analysed.

Predictors of non‐relapse mortality					
Univariate				Multivariate	
Variable	Criterion	*p*‐value	HR (95% CI)	*p*‐value	HR (95% CI)
Age	Continuous	0.303	1.02 (0.98–1.05)		
	Cut >70	0.596	1.28 (0.50–3.27)		
	Cut >75	0.998	NA		
Type of CAR‐T^a^	Tisa‐cel versus Axi‐cel	0.213	0.553 (0.21–1.40)		
Status at CAR‐t	CR‐PR versus SD‐PD	0.962	0.977 (0.38–2.49)		
Gender	Female versus Male	0.646	1.21 (0.52–2.81)		
ECOG	>1	0.309	1.90 (0.54–6.62)		
Stage	I–II versus III–IV	0.654	0.68 (1.13–3.53)		
LDH	Normal versus Elevated	0.091	2.15 (0.88–5.23)		
Previous ASCT		0.728	0.83 (0.31–2.26)		
CIRS	Continuous	0.248	1.08 (0.94–1.25)		
	Cut >6	0.813	1.15 (0.34–3.92)		
HCT‐CI	Continuous	0.962	0.99 (0.81–1.22)		
	Cut >2	0.782	0.88 (0.36–2.14)		
Severe4	0 versus 1	0.580	0.56 (0.07–4.21)		
CRS	>1	0.222	1.66 (0.73–3.77)		
ICANS	>1	0.842	1.11 (0.37–3.28)		
Early ICAHT	>1	**0.027**	**2.75 (1.12–6.75)**	0.074	2.42 (0.91–6.41)
Late ICAHT	>1	0.312	0.56 (0.19–1.69)		
Steroids		0.264	1.66 (0.68–4.05)		
CAR‐HEMATOTOX	Low versus high risk	0.495	1.33 (0.58–3.04)		
m‐EASIX		0.071	1.17 (0.98–1.38)		
	Cut >6.8	**0.049**	**2.80 (1.01–7.84)**	0.224	2.00 (0.65–6.19)

*Note*: Univariable analysis was conducted for all variables. Variables associated with NRM in univariate analysis (*p*‐value up to 0.05, in bold) were then compared in multivariate analysis.

Abbreviations: ASCT, autologous haematopoietic stem cells transplantation; CAT‐T, chimeric antigen receptor T; CRS, Cytokine Release Syndrome; ECOG, Eastern Cooperative Oncology Group; HR, hazard ratio; ICAHT, immune effector cell‐associated haematotoxicity; ICANS, immune effector cell‐associated haematotoxicity; mEASIX, modified endothelial activation and stress index; NRM, non‐relapse mortality; CIRS/Severe4/HCT‐CI see the main text for detailed explanation.

^a^ As liso‐cel counted only for 4% of patients, this specific univariable analysis was conducted only among patients treated with axi‐cel and tisa‐cel.

**FIGURE 1:**
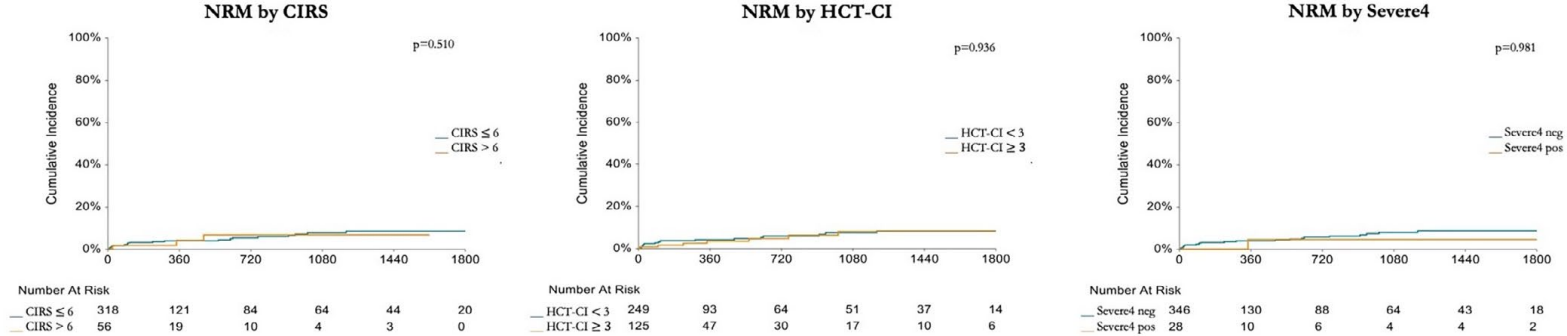
Impact of high comorbidity burden as measured by cumulative illness rating scale, haematopoietic cell transplantation‐specific comorbidity index or Severe4, on the cumulative incidence of non‐relapse mortality (NRM). Regardless of the method of measurement, comorbidity burden did not influence the incidence of NRM.

Our findings are broadly consistent with the existing literature. Major established risk factors known to predict specific toxicities—such as CAR‐T product, disease burden, ECOG performance status, CAR‐HEMATOTOX and mEASIX—were confirmed in our analysis. Real‐world evidence from the CIBMTR experience on liso‐cel showed that, despite over 65% of patients having comorbidities or age that would have excluded them from the registrative trial (cardiac 24%, pulmonary 13%), the incidence of severe toxicities remained similar to trial data (CRS 2%, ICANS 6%)^15^; similar findings are also available for axi‐cel.^16^ Kittai et al. investigated whether higher CIRS scores correlated with increased toxicity and found no association with CRS or ICANS,^10^ nor did Greenbaum et al. with their preliminary CT‐CI CAR‐T comorbidity score.^17^ A recent study of 957 patients in the French DESCAR‐T registry reported a 5% NRM rate post–CAR‐T, mostly due to infections. In that study, individual comorbidities—cardiac, respiratory, hepatic, renal, rheumatologic and gastrointestinal—were not linked to NRM, except for type 2 diabetes. However, cumulative scores like CIRS or HCT‐CI were not assessed.^2^

In conclusion, 7%–33% of the 379 LBCL patients treated with CAR‐T could be considered ‘frail’ based on CIRS, HCT‐CI or Severe4 score. However measured, frail patients had the same risk as fit patients of developing clinically relevant CRS, ICAHT or requiring tocilizumab, steroids or ICU, with a possible higher incidence of severe ICANS in frail patients. We also documented that comorbidity and age did not impact the occurrence of NRM. NRM mostly occurs during the first 2 years, progressively being due to severe CRS/ICANS, infections and secondary myeloid malignancies. These results also align with current evidence from two Phase‐2 trials, the PILOT and the ALYCANTE studies, in which CAR‐T was administered to patients ineligible for stem‐cell transplant.^18^, ^19^ Patients at high risk of NRM are those with a high inflammatory burden and a poor haematopoietic reserve developing early cytopenias.

We acknowledge that the study has some limitations, including the enrolment of patients from only two centres and a relatively short median follow‐up, which limits the focus primarily to short‐ and mid‐term toxicities. However, this is the first report to assess the impact of comorbidity scores on the safety of CAR‐T therapy, and extended follow‐up could further elucidate long‐term safety outcomes. Overall, those findings may be of interest when determining eligibility for therapy, especially when patients are excluded due to comorbidities.

## AUTHOR CONTRIBUTIONS

EG, SS, RDB and CT designed the study. EG, CT, RDB, CC, CB, IDB, MV, IP, FS, PC and AC managed patients and contributed to data collection. EG analysed data and drafted the paper. SH, LM, SS, CB and CT critically revised the paper.

## CONFLICT OF INTEREST STATEMENT

EG received travel grants/speakers' bureau from Novartis, BMS, Kite Gilead, Sanofi. CB has received advisory fees from JNJ, BMS, BeiGene, Abbvie, Cureety (consulting); JNJ, BeiGene (honoraria); Institut Servier (research funding). All other authors declare no competing conflicts of interests.

## ETHICS STATEMENT

The study was conducted in accordance with the Helsinki Declaration and was approved by the local ethics committee (ID 4879 Prot 0020777/22 Amendment 09/2024).

## PATIENTS CONSENT

All participants provided informed consent for the anonymized use of their data.

## Supporting information


Data S1.

## Data Availability

Data available upon request addressed to the corresponding author.
