# Supplementary material for: Frail is not fail: Limited impact of comorbidities on non‐relapse mortality and safety in patients with LBCL treated with CAR‐T
Source: Br J Haematol. 2025 Jun 23;207(2):642–7. doi: 10.1111/bjh.20222 (PMC12378911; doi:10.1111/bjh.20222)
Supplement: Supplementary file 1 — Data S1. [file BJH-207-642-s001.docx]

**SUPPLEMENTARY MATERIAL**

**S1 ASSESSMENT OF COMORBIDITIES**

Regulatory agencies restrict access to CAR-T therapy through well-defined, primarily organ-specific parameters, such as AST/ALT levels, creatinine clearance, and ejection fraction. However, we encountered situations where, despite stringent criteria for certain organs, CAR-T therapy was still prescribed in presence other organ-specific conditions not explicitly restricted by these guidelines. Here we provide a detailed count of comorbidities by organ. Notably, only a few patients had grade 3–4 hepatic or renal impairments (four and one patient, respectively). In contrast, most comorbidities were related to conditions not considered by regulatory agencies but captured by the CIRS and HCT-CI scores, such as visual impairment, rheumatologic diseases, reduced mobility, and previous malignancies. In this context, the criteria set by regulatory agencies appear to assess a general notion of 'fitness,' while comorbidity scores offer a more nuanced depiction of patients’ actual health status.

**CIRS**

The Cumulative Illness Rating Scale (CIRS) assesses the burden of disease across 14 organ systems, assigning scores from 0 (no impairment) to 4 (extremely severe impairment) based on the severity of dysfunction within each system, as follows.

| **Condition** | **Points** | | | | |
| --- | --- | --- | --- | --- | --- |
| Cardiac Diseases (heart only) | 0 | 1 | 2 | 3 | 4 |
| Hypertension (severity grade) | 0 | 1 | 2 | 3 | 4 |
| Vascular Diseases (blood, vessels, bone marrow, spleen, lymph nodes) | 0 | 1 | 2 | 3 | 4 |
| Respiratory Diseases (lungs, bronchi, trachea) | 0 | 1 | 2 | 3 | 4 |
| Diseases of the Eye, Ear, Nose, Throat, Larynx | 0 | 1 | 2 | 3 | 4 |
| Endocrine-Metabolic Diseases (diabetes, infections, sepsis, toxic states) | 0 | 1 | 2 | 3 | 4 |
| Upper Gastrointestinal Diseases (esophagus, stomach, duodenum, biliary tree, pancreas) | 0 | 1 | 2 | 3 | 4 |
| Lower Gastrointestinal Diseases (intestines, hernias) | 0 | 1 | 2 | 3 | 4 |
| Liver Diseases | 0 | 1 | 2 | 3 | 4 |
| Kidney Diseases | 0 | 1 | 2 | 3 | 4 |
| Genitourinary Diseases (ureters, bladder, urethra, prostate, genitalia) | 0 | 1 | 2 | 3 | 4 |
| Diseases of Muscles, Skeleton, and Skin | 0 | 1 | 2 | 3 | 4 |
| Diseases of the Peripheral and Central Nervous System (excluding dementia) | 0 | 1 | 2 | 3 | 4 |
| Psychiatric-Behavioral Diseases (dementia, depression, anxiety, agitation, psychosis) | 0 | 1 | 2 | 3 | 4 |

• **0**: No disease.

• **1**: Mild disease, no significant functional impact.

• **2**: Moderate disease, requires treatment, some functional impact.

• **3**: Severe disease, significant functional impact, intensive treatment required.

• **4**: Very severe disease, life-threatening, requires invasive treatment or frequent hospitalization; malignant neoplasia.

This scale captures both chronic and acute conditions (Salvi et al., J Am Geriatr Soc 2008). Tumors are evaluated according to their size, invasiveness, and metastatic spread, with higher scores reflecting more advanced or widespread malignancies. The total CIRS score provides a comprehensive measure of a patient’s comorbidity burden, offering valuable prognostic insight and informing clinical decisions.

In this study, data were extracted from electronic medical records.

Hematologic cancer was excluded from the cumulative score, as it was common to all patients.

Overall distribution of patients according to the total CIRS score was as follows:


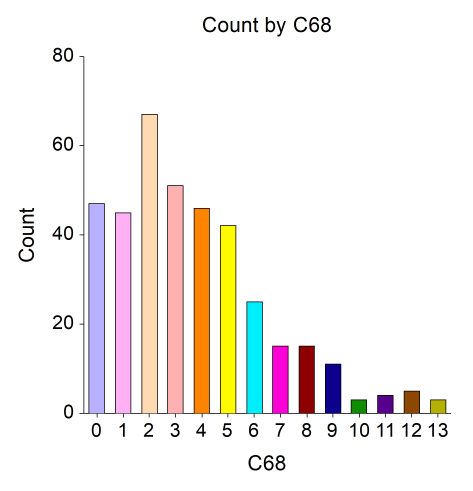


X axis: cumulative CIRS score

Y axis: number of patients

**CIRS score Nr Patients**

0 47

1 45

2 67

3 51

4 46

5 42

6 25

7 15

8 15

9 11

10 3

11 4

12 5

13 3

The distribution of organ-specific comorbidities Scored/Graded 0-to-4 among the 379 patients in the study was as follows (Table and histogram):

| **Score** | **Heart** | **Hypertension** | **Vascular/Hemat** | **Lung** | **Senses** | **Endocrinology** | **UpperGI** | **LowerGI** | **Liver** | **Kidney** | **Genit-Urinary** | **Muscle-Skel** | **Neurologic** | **Psychiatric** |
| --- | --- | --- | --- | --- | --- | --- | --- | --- | --- | --- | --- | --- | --- | --- |
| 0 | 326 | 297 | 303 | 237 | 349 | 276 | 353 | 324 | 317 | 358 | 319 | 297 | 356 | 370 |
| 1 | 21 |  | 21 | 96 | 18 | 51 | 5 | 32 | 40 | 7 | 22 | 40 | 9 | 7 |
| 2 | 25 | 80 | 31 | 33 | 9 | 51 | 18 | 20 | 18 | 13 | 25 | 32 | 13 | 2 |
| 3 | 7 | 2 | 20 | 13 | 2 | 1 | 3 | 3 | 2 | 1 | 11 | 10 | 1 |  |
| 4 |  |  | 4 |  | 1 |  |  |  | 2 |  | 2 |  |  |  |


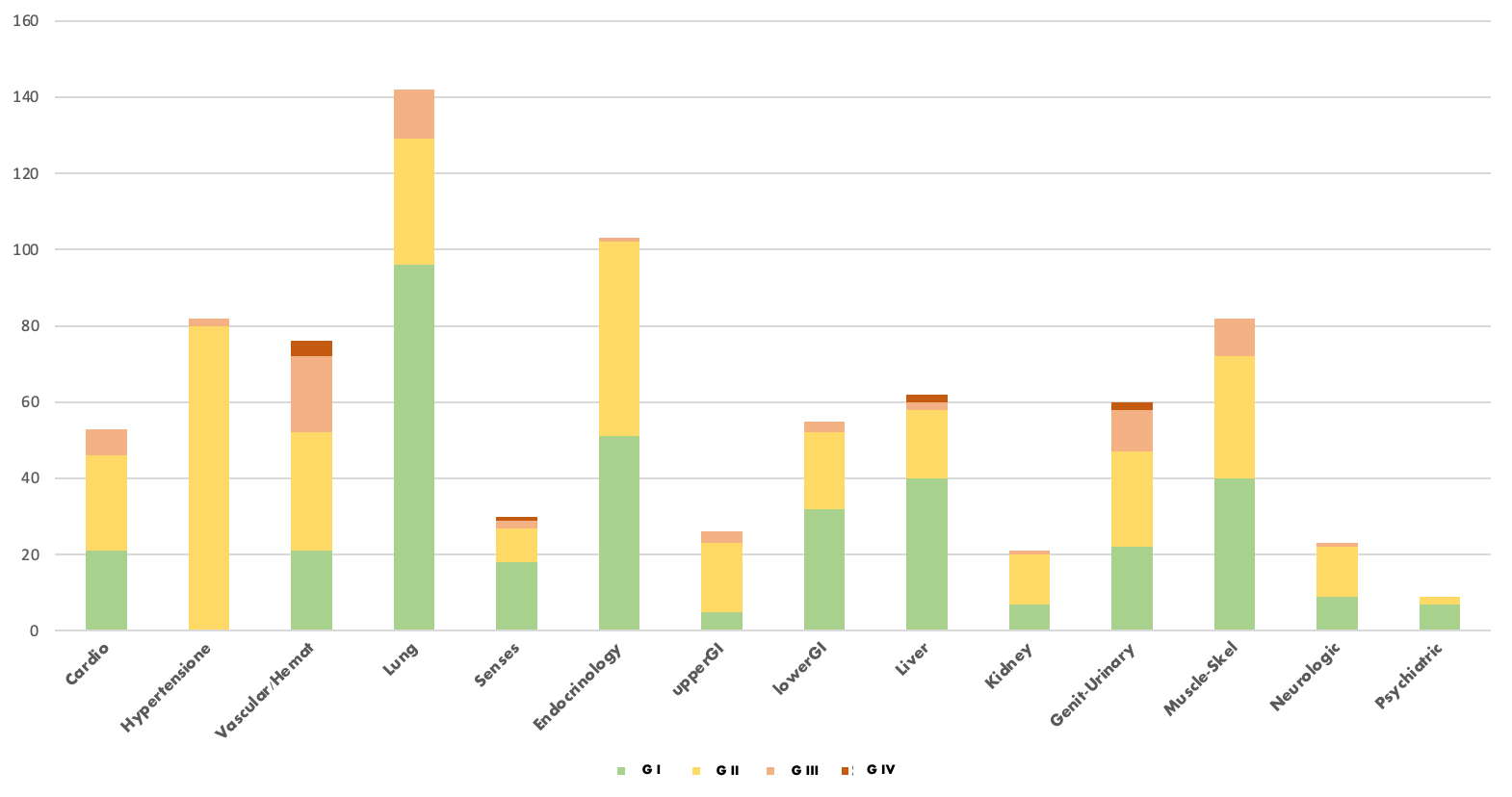


**HCT-CI**

The Hematopoietic Cell Transplantation-specific Comorbidity Index (HCT-CI) is a tool designed to predict non-relapse mortality (NRM) and overall outcomes in patients undergoing hematopoietic stem cell transplantation (HSCT). It evaluates 14 organ systems, assigning scores from 0 to 3 based on the severity of pre-existing comorbidities. Higher scores reflect greater organ dysfunction and correlate with increased risk of complications and mortality post-transplant. The index includes conditions such as cardiovascular disease, pulmonary dysfunction, and liver impairment.

| **Condition** | **0 (None)** | **1 (Mild)** | **2 (Moderate)** | **3 (Severe)** |
| --- | --- | --- | --- | --- |
| **History of Arrhythmia** | None | Atrial fib/flutter, sick sinus syndrome, or ventricular arrhythmias | - | - |
| **Cardiac Disease (CAD, CHF, MI, EF ≤50%)** | None | CAD, CHF, MI, or EF ≤50% | - | Valvular disease (except mitral prolapse) |
| **Inflammatory Bowel Disease** | None | Crohn disease or ulcerative colitis | - | - |
| **Diabetes** | None or diet-controlled | Treated with insulin or oral hypoglycemics | - | - |
| **Cerebrovascular Disease (CVA, TIA)** | None | CVA or TIA | - | - |
| **Psychiatric Disturbance** | None | Depression or anxiety requiring psych consult or treatment | - | - |
| **Hepatic Dysfunction** | None | Chronic hepatitis (bilirubin > ULN to 1.5× ULN, or AST/ALT > ULN to 2.5× ULN) | Liver cirrhosis (bilirubin >1.5× ULN, or AST/ALT 2.5× ULN) | - |
| **Obesity (BMI ≥35 kg/m²)** | No | Yes | - | - |
| **Infection** | None or abx only on day 0 | Requiring continuation of abx after day 0 | - | - |
| **Rheumatologic Disease** | None | Systemic lupus erythematosus, rheumatoid arthritis, polymyositis, mixed connective tissue disorder, or polymyalgia rheumatica | - | - |
| **Peptic Ulcer** | None or not requiring treatment | Requiring treatment | - | - |
| **Renal Dysfunction** | None or serum Cr ≤2 mg/dL (177 µmol/L), not on dialysis, and no prior renal transplant | Serum Cr >2 mg/dL (177 µmol/L), on dialysis, or prior renal transplant | - | - |
| **Pulmonary Dysfunction** | None or mild | DLCO/FEV₁ 66%–80%, or dyspnea on slight activity | DLCO/FEV₁ ≤65% or dyspnea at rest or requiring oxygen | - |
| **Prior Solid Tumor** | None or nonmelanoma skin cancer | Treated at any point in the patient’s history | - | - |

CAD Coronary Artery Disease; CHF Congestive Heart Failure; MI Myocardial Infarction; EF Ejection Fraction; CVA Cerebrovascular Accident; TIA Transient Ischemic Attack; ULN Upper Limit of Normal; DLCO Diffusing Capacity of the Lungs for Carbon Monoxide; FEV1 Forced Expiratory Volume in 1 second

By quantifying comorbidities, the HCT-CI helps stratify patients by risk, guiding treatment decisions and transplant eligibility.

Overall cumulative HCT-CI was distributed as follows:


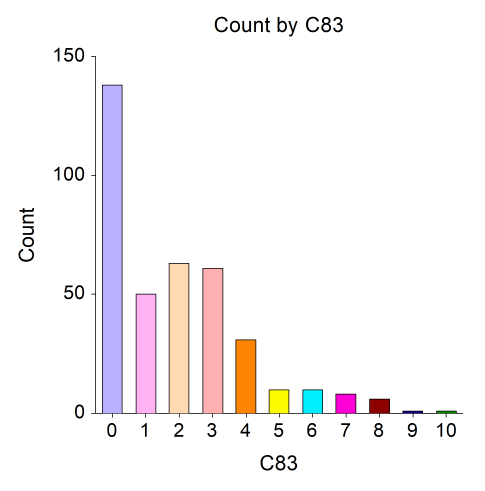


X axis: cumulative HCT-CI score

Y axis: number of patients

**HCT-CI Nr Patients**

0 138

1 50

2 63

3 61

4 31

5 10

6 10

7 8

8 6

9 1

10 1

In our 379 patients, HCT-CI was distributed as follows (cases per points assigned)

| Points | Arrythmia | Cardiac | IBD | Diabetes | Cerebrovascular | Psychiatric | Liver | Obesity | Infections | Autoimmune | Peptic Ulcer | Kidney | Lungs | Prior Tumor |
| --- | --- | --- | --- | --- | --- | --- | --- | --- | --- | --- | --- | --- | --- | --- |
| 1 | 20 | 37 | 2 | 32 | 7 | 7 | 13 | 10 | 31 | 7 |  | 1 |  |  |
| 2 |  |  |  |  |  | 13 | 12 |  |  |  | 6 | 5 | 100 |  |
| 3 |  | 1 |  |  |  |  |  |  |  |  |  |  | 42 | 43 |


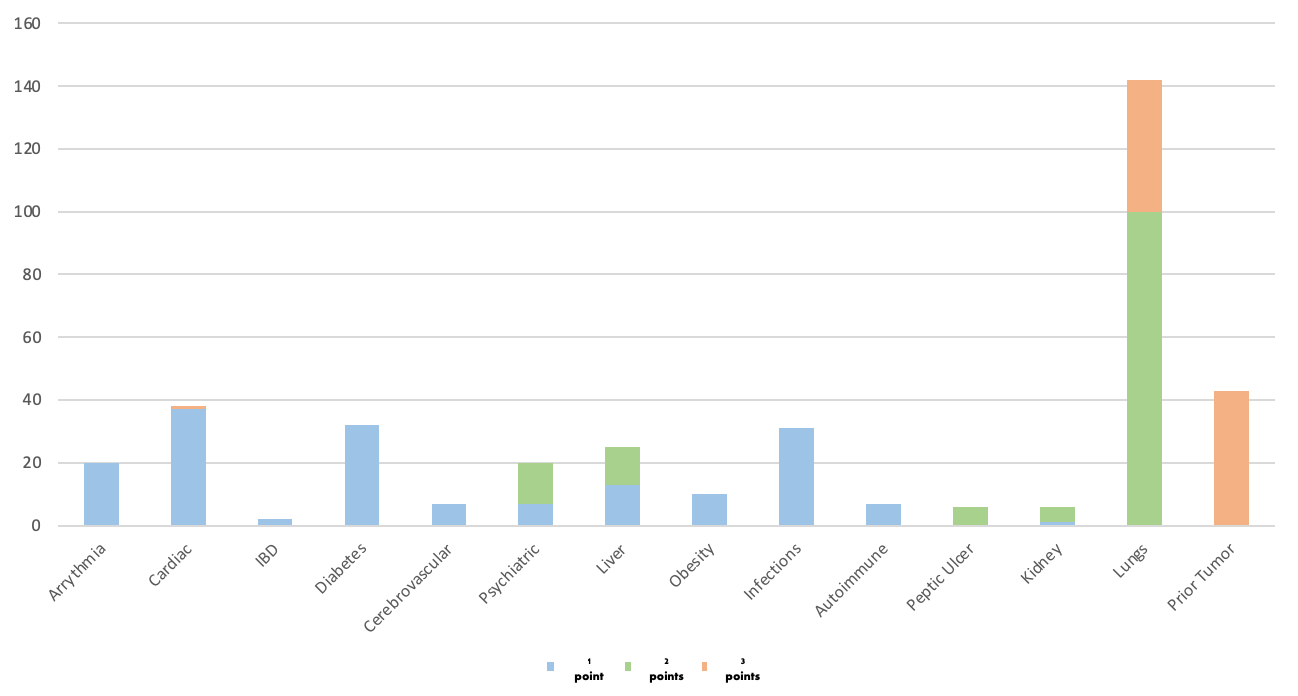


*References*

*Salvi F, Miller MD, Grilli A, Giorgi R, Towers AL, Morichi V, Spazzafumo L, Mancinelli L, Espinosa E, Rappelli A, Dessì-Fulgheri P. A manual of guidelines to score the modified cumulative illness rating scale and its validation in acute hospitalized elderly patients. J Am Geriatr Soc. 2008 Oct;56(10):1926-31. doi: 10.1111/j.1532-5415.2008.01935.x. Epub 2008 Sep 22. PMID: 18811613.*

*Sorror ML, Maris MB, Storb R, Baron F, Sandmaier BM, Maloney DG, Storer B. Hematopoietic cell transplantation (HCT)-specific comorbidity index: a new tool for risk assessment before allogeneic HCT. Blood. 2005 Oct 15;106(8):2912-9. doi: 10.1182/blood-2005-05-2004. Epub 2005 Jun 30. PMID: 15994282; PMCID: PMC1895304.*

**S2 STROBE (Strengthening the Reporting of Observational Studies in Epidemiology) Statement—checklist of items that should be included in reports of observational studies**

|  | | Item No | Recommendation |  |
| --- | --- | --- | --- | --- |
| **Title and abstract** | | 1 | 1. Indicate the study’s design with a commonly used term in the title or the abstract   Multicenter observational cohort study on collected data as stated in the abstract |  |
|  |  |  | 1. Provide in the abstract an informative and balanced summary of what was done and what was found   Provided in the abstract |  |
| Introduction | | | |  |
| Background/rationale | | 2 | Explain the scientific background and rationale for the investigation being reported  Provided in introductive section |  |
| Objectives | | 3 | State specific objectives, including any prespecified hypotheses  Provided in Aims section |  |
| Methods | | | |  |
| Study design | | 4 | Present key elements of study design early in the paper  Inclided in Aims section |  |
| Setting | | 5 | Describe the setting, locations, and relevant dates, including periods of recruitment, exposure, follow-up, and data collection  Included in Patients and Methods section |  |
| Participants | | 6 | (*a*) *Cohort study*—Give the eligibility criteria, and the sources and methods of selection of participants. Describe methods of follow-up Included in Patients and Methods section  *Case-control study*—Give the eligibility criteria, and the sources and methods of case ascertainment and control selection. Give the rationale for the choice of cases and controls  *Cross-sectional study*—Give the eligibility criteria, and the sources and methods of selection of participants |  |
|  |  |  | (*b*) *Cohort study*—For matched studies, give matching criteria and number of exposed and unexposed  *Case-control study*—For matched studies, give matching criteria and the number of controls per case |  |
| Variables | | 7 | Clearly define all outcomes, exposures, predictors, potential confounders, and effect modifiers. Give diagnostic criteria, if applicable Included in Patients and Methods section |  |
| Data sources/ measurement | | 8* | For each variable of interest, give sources of data and details of methods of assessment (measurement). Describe comparability of assessment methods if there is more than one group Included in Patients and Methods section and, for determination of CIRS, HCTCI and Severe4, provided in Supplementary materials |  |
| Bias | | 9 | Describe any efforts to address potential sources of bias  Discussed the positive selection of patients in the Discussion |  |
| Study size | | 10 | Explain how the study size was arrived at NA |  |
| Quantitative variables | | 11 | Explain how quantitative variables were handled in the analyses. If applicable, describe which groupings were chosen and why Included in Patients and Methods section |  |
| Statistical methods | | 12 | (*a*) Describe all statistical methods, including those used to control for confounding Included in Patients and Methods section |  |
|  |  |  | (*b*) Describe any methods used to examine subgroups and interactions |  |
|  |  |  | (*c*) Explain how missing data were addressed |  |
|  |  |  | (*d*) *Cohort study*—If applicable, explain how loss to follow-up was addressed  *Case-control study*—If applicable, explain how matching of cases and controls was addressed  *Cross-sectional study*—If applicable, describe analytical methods taking account of sampling strategy |  |
|  |  |  | (*e*) Describe any sensitivity analyses NA |  |
| Results | | | | |
| Participants | | 13* | (a) Report numbers of individuals at each stage of study—eg numbers potentially eligible, examined for eligibility, confirmed eligible, included in the study, completing follow-up, and analysed Provided in the Results section | |
|  |  |  | (b) Give reasons for non-participation at each stage Provided in the Results section | |
|  |  |  | (c) Consider use of a flow diagram | |
| Descriptive data | | 14* | (a) Give characteristics of study participants (eg demographic, clinical, social) and information on exposures and potential confounders Provided in Tables | |
|  |  |  | (b) Indicate number of participants with missing data for each variable of interest Collectable from Tables | |
|  |  |  | (c) *Cohort study*—Summarise follow-up time (eg, average and total amount) Reported in Results | |
| Outcome data | | 15* | *Cohort study*—Report numbers of outcome events or summary measures over time Provided in Results | |
|  |  |  | *Case-control study—*Report numbers in each exposure category, or summary measures of exposure | |
|  |  |  | *Cross-sectional study—*Report numbers of outcome events or summary measures | |
| Main results | | 16 | (*a*) Give unadjusted estimates and, if applicable, confounder-adjusted estimates and their precision (eg, 95% confidence interval). Make clear which confounders were adjusted for and why they were included Provided in Results | |
|  |  |  | (*b*) Report category boundaries when continuous variables were categorized | |
|  |  |  | (*c*) If relevant, consider translating estimates of relative risk into absolute risk for a meaningful time period NA | |
| Other analyses | | 17 | Report other analyses done—eg analyses of subgroups and interactions, and sensitivity analyses | |
| Discussion | | | | |
| Key results | | 18 | Summarise key results with reference to study objectives Done in conclusions | |
| Limitations | | 19 | Discuss limitations of the study, taking into account sources of potential bias or imprecision. Discuss both direction and magnitude of any potential bias Selection of patients discussed in the Discussion | |
| Interpretation | | 20 | Give a cautious overall interpretation of results considering objectives, limitations, multiplicity of analyses, results from similar studies, and other relevant evidence Done in Discussion | |
| Generalisability | | 21 | Discuss the generalisability (external validity) of the study results | |
| Other information | | | | |
| Funding | | 22 | Give the source of funding and the role of the funders for the present study and, if applicable, for the original study on which the present article is based Provided in Acknoledgements | |

*Reference: von Elm E, Altman DG, Egger M, Pocock SJ, Gøtzsche PC, Vandenbroucke JP; STROBE Initiative. The Strengthening the Reporting of Observational Studies in Epidemiology (STROBE) statement: guidelines for reporting observational studies. Lancet. 2007 Oct 20;370(9596):1453–7. doi:10.1016/S0140-6736(07)61602-X.*

**S3 STATISTICAL ANALYSIS**

Categorical and continuous variables were analyzed using classical descriptive statistics, specifically Fisher’s exact test or the T-test. For Progression-Free Survival (PFS) and Non-Relapse Mortality (NRM), time was measured from the day of CAR-T infusion, and data were analyzed using Cumulative Incidence and Gray’s test (with relapse and mortality as competitive events). Inferential statistics were conducted as follows: correlations between categorical/continuous variables and binary outcomes or survival were assessed using logistic regression or the Cox regression model, respectively. Univariable analysis considered a p-value of up to 5% as significant. Multivariate analysis was performed on variables with significant univariable correlations.

Statistical analysis was conducted using NCSS 2020 (NCSS, LLC, Kaysville, Utah, USA, ncss.com/software/ncss).

**S4 IMPACT OF COMORBIDITIES ON TOXICITIES**

The incidence of toxicities by comorbidity group is represented in pie-charts. For ICAHT, only patients with no further relapse were considered.


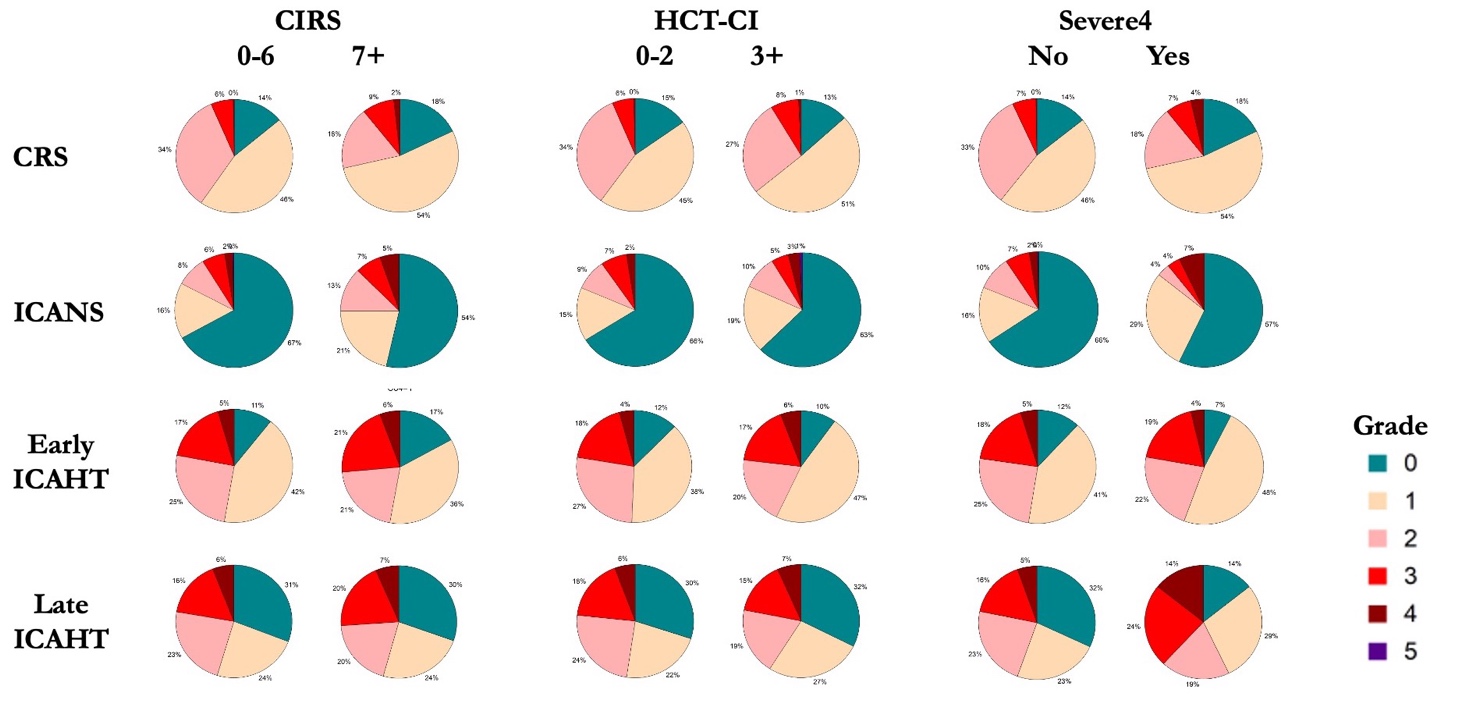


To minimize potential confounding in the analysis of comorbidity scores and their association with CRS, ICANS, and ICAHT, we conducted multivariable logistic regression analyses. As shown in Table 1 of the main manuscript, patients with high and low comorbidity burdens were largely comparable, with the main differences being a higher proportion of elderly and male patients in the high-comorbidity group.

We analyzed the association of each characteristic of the study population with CRS, ICANS, early ICAHT, and late ICAHT using univariable analysis, alongside comorbidity burden assessed by CIRS, HCT-CI, or Severe4. Variables found to be associated with the specific outcome (p < 0.05 in univariable analysis) were included in a multivariable model, which always incorporated the comorbidity score.

**Multivariate analysis for prediction of CRS (grade 2-4), ICANS (grade 2-5), and early or late ICAHT (grade 2-4) comparing CIRS>6 with the main characteristics.**

|  | **CRS 2-4** | | **ICANS 2-5** | | **Early ICAHT 2-4** | | **Late ICAHT 2-4** | |
| --- | --- | --- | --- | --- | --- | --- | --- | --- |
|  | **Univariate** | **Multivariate** | **Univariate** | **Multivariate** | **Univariate** | **Multivariate** | **Univariate** | **Multivariate** |
|  | **OR (95% CI), P value** | **OR (95% CI), P value** | **OR (95% CI), P value** | **OR (95% CI), P value** | **OR (95% CI), P value** | **OR (95% CI), P value** | **OR (95% CI), P value** | **OR (95% CI), P value** |
| **CIRS >6** | 0.63 (0.36-1.09), p 0.101 | 0.73 (0.36-1.47), p 0.370 | 1.47 (0.85-2.54), p 0.163 | **2.48 (1.16-5.28), p 0.018** | 0.99 (0.60-1.64), p 0.991 | 0.87 (0.44-1.70), p 0.689 | 1.01 (0.59-1.72) p 0.969 | 0.92 (0.49-1.74) p 0.81 |
| **Male Gender** | **0.79 (0.66-0.95), p 0.013** | 0.84 (0.62-1-14), p 0.271 | 0.98 (0.79-1.22), p 0.890 |  | 1.03 (0.87-1.23), p 0.681 |  | 0.86 (0.71-1.03) p 0.117 |  |
| **Age >70** | 0.82 (0.56-1.19), p 0.297 |  | 1.45 (0.98-2.14), p 0.062 |  | 0.93 (0.64-1.34), p 0.700 |  | 1.05 (0.72-1.55) p 0.766 |  |
| **Diagnosis PMBL vs DLBCL** | 1.78 (0.70- 4.52), p 0.220 |  | 0.94 (0.27-3.20), p 0.930 |  | 1.59 (0.62-4.09), p 0.331 |  | 0.90 (0.32-2.53) p 0.845 |  |
| **tFL vs DLBCL** | **0.48 (0.29-0.80), p 0.004** | 0.75 (0.40-1-40), p 0.369 | **1.69 (0.08-0.33), p<0001** | **0.24 (0.09-0.60), p 0.002** | 1.00 (0.61-1.63). p 1.00 |  | 0.83 (0.49-1.41) p 0.508 |  |
| **Type of CAR-T Liso-Cel vs Axi-Cel** | **0.11 (0.01-0.85), p 0.034** | **0.09 (0.01-0.76), p 0.026** | 0.31 (0.04-2.36), p 0.261 | **0.08 (0.01-0.67), p 0.019** | 0.44 (0.14-1.39), p 0.166 | **0.18 (0.03-0.89), p 0.036** | 0.80 (0.29-2.19) p 0.667 | 0.76 (0.27-2.11) p 0.603 |
| **Type of CAR-T Tisa-Cel vs Axi-Cel** | **0.53 (0.37- 0.74), p<0.001** | 0.77 (0.49-1.23), p 0.284 | **0.11 (0.06-1.19), p<0.001** | **0.14 (0.07-0.27), p<0.001** | **0.51 (0.35-0.72), p<0.001** | **0.27 (0.16-0.46), p<0.001** | **0.62 (0.43-0.90) p 0.013** | **0.52 (0.33-0.80) p 0.003** |
| **Status before CAR-T (SD/PD vs CR-PR)** | 1.10 (0.97-1.25), p 0.121 |  | 1.05 (0.89-1.23), p 0.545 |  | 0.92 (0.80-1.05), p 0.259 |  | 1.04 (0.90-1.20) p 0.561 |  |
| **ECOG >1** | **2.13 (1.27-3.57), p 0.003** | **2.05 (1.04-4.03), p 0.035** | **2.79 (1.68-4.62), p<0.001** | 1.46 (0.69-3.10), p 0.318 | **2.07 (1.19-3.60), p 0.009** | 1.52 (0.73-3.16), p 0.254 | 1.35 (0.71-2.55) p 0.351 |  |
| **LDH elevated** | 1.16 (0.95-1.40), p 0.123 |  | 1.06 (0.83-1.35), p 0.615 |  | **1.41 (1.15-1.73), p<0.001** | 1.16 (0.68-1.96), p 0.569 | 1.10 (0.88-1.39) p 0.374 |  |
| **Stage III-IV** | 1.05 (0.93-1.18), p 0.387 |  | 1.04 (0.90-1.20), p 0.535 |  | **1.19 (1.06-1.34), p 0.003** | 1.09 (0.68-1.76), p 0.698 | 1.00 (0.87-1.15) p 0.928 |  |
| **CAR-HEMATOTOX High** | 1.03 (0.84-.26), p 0.727 |  | 1.14 (0.90-1.45), p 0.249 |  | **1.56 (1.27-1.91), p<0.001** | **1.93 (1.13-3.30), p 0.015** | **1.38 (1.10- 1.72) p 0.004** | **1.47 (1.01-2.15) p 0.043** |
| **mEASIX >6.8** | **1.85 (1.12-3.04), p 0.014** | 1.80 (0.95-3.39), p 0.068 | **2.20 (1.31-3.68), p 0.002** | 1.25 (0.60-2.59), p 0.546 | **1.71 (1.01-2.88), p 0.043** | 1.15 (0.55-2.40), p 0.700 | 1.41 (0.76-2.62) p 0.266 |  |

**Multivariate analysis for prediction of CRS (grade 2-4), ICANS (grade 2-5), and early or late ICAHT (grade 2-4) comparing HCT-CI≥3 with the main characteristics.**

|  | **CRS 2-4** | | **ICANS 2-5** | | **Early ICAHT 2-4** | | **Late ICAHT 2-4** | |
| --- | --- | --- | --- | --- | --- | --- | --- | --- |
|  | **Univariate** | **Multivariate** | **Univariate** | **Multivariate** | **Univariate** | **Multivariate** | **Univariate** | **Multivariate** |
|  | **OR (95% CI), P value** | **OR (95% CI), P value** | **OR (95% CI), P value** | **OR (95% CI), P value** | **OR (95% CI), P value** | **OR (95% CI), P value** | **OR (95% CI), P value** | **OR (95% CI), P value** |
| **HCT-CI≥3** | 0.89 (0.66-1.19), p 0.446 | 1.00 (0.59-1.67), p 0.99 | 0.98 (0.68-1.42), p 0.944 | **1.86 (1.13-3.04), p 0.013** | 0.84 (0.62-1.13), p 0.252 | 0.70 (0.44-1.13), p 0.148 | 0.83 (0.60-1.14) p 0.266 | 0.66 (0.42-1.05) p 0.084 |
| **Gender** | **0.79 (0.66-0.95), p 0.013** | 0.80 (0.57-1.14), p 0.231 | 0.98 (0.79-1.22), p 0.890 |  | 1.03 (0.87-1.23), p 0.681 |  | 0.86 (0.71-1.03) p 0.117 |  |
| **Age >70** | 0.82 (0.56-1.19), p 0.297 |  | 1.45 (0.98-2.14), p 0.062 |  | 0.93 (0.64-1.34), p 0.700 |  | 1.05 (0.72-1.55) p 0.766 |  |
| **Diagnosis PMBL vs DLBCL** | 1.78 (0.70- 4.52), p 0.220 |  | 0.94 (0.27-3.20), p 0.930 |  | 1.59 (0.62-4.09), p 0.331 |  | 0.90 (0.32-2.53) p 0.845 |  |
| **tFL vs DLBCL** | **0.48 (0.29-0.80), p 0.004** | 0.72 (0.39-1.35), p 0.313 | **1.69 (0.08-0.33), p<0001** | **0.27 (0.11-0.69), p 0.006** | 1.00 (0.61-1.63). p 1.00 |  | 0.83 (0.49-1.41) p 0.508 |  |
| **Type of CAR-T Liso-Cel vs Axi-Cel** | **0.11 (0.01-0.85), p 0.034** | **0.09 (0.01-0.73), p 0.024** | 0.31 (0.04-2.36), p 0.261 | **0.09 (001-0.76), p 0.026** | 0.44 (0.14-1.39), p 0.166 | **0.17 (0.03-0.87), p 0.033** | 0.80 (0.29-2.19) p 0.667 | 0.82 (0.29-2.28) p 0.705 |
| **Type of CAR-T Tisa-Cel vs Axi-Cel** | **0.53 (0.37- 0.74), p<0.001** | 0.76 (0.48-1.21), p 0.262 | **0.11 (0.06-1.19), p<0.001** | **0.168 (0.08-0.32), p<0.001** | **0.51 (0.35-0.72), p<0.001** | **0.27 (0.16-0.47), p<0.001** | **0.62 (0.43-0.90) p 0.013** | **0.57 (0.36-0.89) p 0.014** |
| **Status before CAR-T (SD/PD vs CR-PR)** | 1.10 (0.97-1.25), p 0.121 |  | 1.05 (0.89-1.23), p 0.545 |  | 0.92 (0.80-1.05), p 0.259 |  | 1.04 (0.90-1.20) p 0.561 |  |
| **ECOG >1** | **2.13 (1.27-3.57), p 0.003** | **1.97 (1.01-3.83), p 0.045** | **2.79 (1.68-4.62), p<0.001** | 1.60 (0.76-3.36), p 0.221 | **2.07 (1.19-3.60), p 0.009** | 1.49 (0.72-3.08), p 0.273 | 1.35 (0.71-2.55) p 0.351 |  |
| **LDH elevated** | 1.16 (0.95-1.40), p 0.123 |  | 1.06 (0.83-1.35), p 0.615 |  | **1.41 (1.15-1.73), p<0.001** | 1.20 (0.71-2.03), p 0.493 | 1.10 (0.88-1.39) p 0.374 |  |
| **Stage III-IV** | 1.05 (0.93-1.18), p 0.387 |  | 1.04 (0.90-1.20), p 0.535 |  | **1.19 (1.06-1.34), p 0.003** | 1.18 (0.73-1.93), 0.485 | 1.00 (0.87-1.15) p 0.928 |  |
| **CAR-HEMATOTOX High** | 1.03 (0.84-.26), p 0.727 |  | 1.14 (0.90-1.45), p 0.249 |  | **1.56 (1.27-1.91), p<0.001** | **2.01 (1.17-3.44), p 0.010** | **1.38 (1.10- 1.72) p 0.004** | **1.59 (1.08-2.34) p 0.017** |
| **mEASIX** | **1.85 (1.12-3.04), p 0.014** | 1.82 (0.96-3.43), p 0.064 | **2.20 (1.31-3.68), p 0.002** | 1.30 (0.62-2.71), p 480 | **1.71 (1.01-2.88), p 0.043** | 1.08 (0.51-2.28), p 0.830 | 1.41 (0.76-2.62) p 0.266 |  |

**Multivariate analysis for prediction of CRS (grade 2-4), ICANS (grade 2-5), and early or late ICAHT (grade 2-4) comparing Severe4 with the main characteristics.**

|  | **CRS 2-4** | | **ICANS 2-5** | | **Early ICAHT 2-4** | | **Late ICAHT 2-4** | |
| --- | --- | --- | --- | --- | --- | --- | --- | --- |
|  | **Univariate** | **Multivariate** | **Univariate** | **Multivariate** | **Univariate** | **Multivariate** | **Univariate** | **Multivariate** |
|  | **OR (95% CI), P value** | **OR (95% CI), P value** | **OR (95% CI), P value** | **OR (95% CI), P value** | **OR (95% CI), P value** | **OR (95% CI), P value** | **OR (95% CI), P value** | **OR (95% CI), P value** |
| **Severe4** | 0.63 (0.28-1.40), p 0.262 | 0.61 (0.24-1.59), p 0.322 | 0.73 (0.26-2.05), p 0.559 | 1.30 (0.38-4.43), p 0.672 | 0.89 (0.43-1.85), p 0.762 | 0.78 (0.32-1.89), p 0.592 | 1.60 (0.69-1.69) p 0.267 | 1.31 (0.52-3.29) p 0.560 |
| **Gender** | **0.79 (0.66-0.95), p 0.013** | 0.85 (0.62-1.15), p 0.307 | 0.98 (0.79-1.22), p 0.890 |  | 1.03 (0.87-1.23), p 0.681 |  | 0.86 (0.71-1.03) p 0.117 |  |
| **Age >70** | 0.82 (0.56-1.19), p 0.297 |  | 1.45 (0.98-2.14), p 0.062 |  | 0.93 (0.64-1.34), p 0.700 |  | 1.05 (0.72-1.55) p 0.766 |  |
| **Diagnosis PMBL vs DLBCL** | 1.78 (0.70- 4.52), p 0.220 |  | 0.94 (0.27-3.20), p 0.930 |  | 1.59 (0.62-4.09), p 0.331 |  | 0.90 (0.32-2.53) p 0.845 |  |
| **tFL vs DLBCL** | **0.48 (0.29-0.80), p 0.004** | 0.74 (0.39-1.38), p 0.349 | **1.69 (0.08-0.33), p<0001** | **0.25 (0.10-0..61), p 0.02** | 1.00 (0.61-1.63). p 1.00 |  | 0.83 (0.49-1.41) p 0.508 |  |
| **Type of CAR-T Liso-Cel vs Axi-Cel** | **0.11 (0.01-0.85), p 0.034** | **0.09 (0.01-0.72), p 0.023** | 0.31 (0.04-2.36), p 0.261 | **0.07 (0.01-0.61), p 0.015** | 0.44 (0.14-1.39), p 0.166 | **0.17 (0.03-0.87), p 0.034** | 0.80 (0.29-2.19) p 0.667 | 0.76 (0.27-2.10) p 0.597 |
| **Type of CAR-T Tisa-Cel vs Axi-Cel** | **0.53 (0.37- 0.74), p<0.001** | 0.76 (0.48-1.21), p 0.260 | **0.11 (0.06-1.19), p<0.001** | **0.14 (0.07-0.27), p<0.001** | **0.51 (0.35-0.72), p<0.001** | **0.27 (0.16-0.46), p<0.001** | **0.62 (0.43-0.90) p 0.013** | **0.51 (0.33-0.79) p 0.002** |
| **Status before CAR-T (SD/PD vs CR-PR)** | 1.10 (0.97-1.25), p 0.121 |  | 1.05 (0.89-1.23), p 0.545 |  | 0.92 (0.80-1.05), p 0.259 |  | 1.04 (0.90-1.20) p 0.561 |  |
| **ECOG >1** | **2.13 (1.27-3.57), p 0.003** | **2.00 (1.02-3.89), p 0.041** | **2.79 (1.68-4.62), p<0.001** | 1.37 (0.65-2.88), p 0.397 | **2.07 (1.19-3.60), p 0.009** | 1.49 (0.72-3.08), p 0.276 | 1.35 (0.71-2.55) p 0.351 |  |
| **LDH elevated** | 1.16 (0.95-1.40), p 0.123 |  | 1.06 (0.83-1.35), p 0.615 |  | **1.41 (1.15-1.73), p<0.001** | 1.17 (0.69-1.98), p 0.544 | 1.10 (0.88-1.39) p 0.374 |  |
| **Stage III-IV** | 1.05 (0.93-1.18), p 0.387 |  | 1.04 (0.90-1.20), p 0.535 |  | **1.19 (1.06-1.34), p 0.003** | 1.09 (0.68-1.74), p 0.716 | 1.00 (0.87-1.15) p 0.928 |  |
| **CAR-HEMATOTOX High** | 1.03 (0.84-.26), p 0.727 |  | 1.14 (0.90-1.45), p 0.249 |  | **1.56 (1.27-1.91), p<0.001** | **1.92 (1.13-3.27), p 0.015** | **1.38 (1.10- 1.72) p 0.004** | 1.44 (0.99-2.10) p 0.054 |
| **mEASIX** | **1.85 (1.12-3.04), p 0.014** | 1.82 (0.96-3.43), p 0.064 | **2.20 (1.31-3.68), p 0.002** | 1.25 (0.60-2.60), p 0.548 | **1.71 (1.01-2.88), p 0.043** | 1.16 (0.55-2.42), p 0.682 | 1.41 (0.76-2.62) p 0.266 |  |

Overall, comorbidity burden was not associated with CAR-T-related toxicities, except for a possible association with an increased risk of ICANS, observed with 2 out of 3 comorbidity scores.

CRS was independently predicted by higher ECOG and mEASIX scores, while patients treated with liso-cel had a lower risk.

ICANS occurred less frequently in patients treated with liso-cel or tisa-cel compared to axi-cel, as well as in those with tFL compared to DLBCL.

ICAHT was strongly predicted by CAR-HEMATOTOX, with lower incidence observed in patients treated with tisa-cel (and liso-cel for early ICAHT).

**S5 IMPACT OF COMORBIDITIES ON ELDERLY POPULATION AGED >= 70 years**

Overall, patients aged more than 70 years were 94 (24.8%).

Patients aged more then 70 with high comorbidity burden were 23.4%, 8.5%, and 36.1% if considered with CIRS >6, Severe 4, or HCT-CI >=3, respectively.

Also in this elderly population, the cumulative incidence of NRM was not different when comparing frail patients with the others.

**
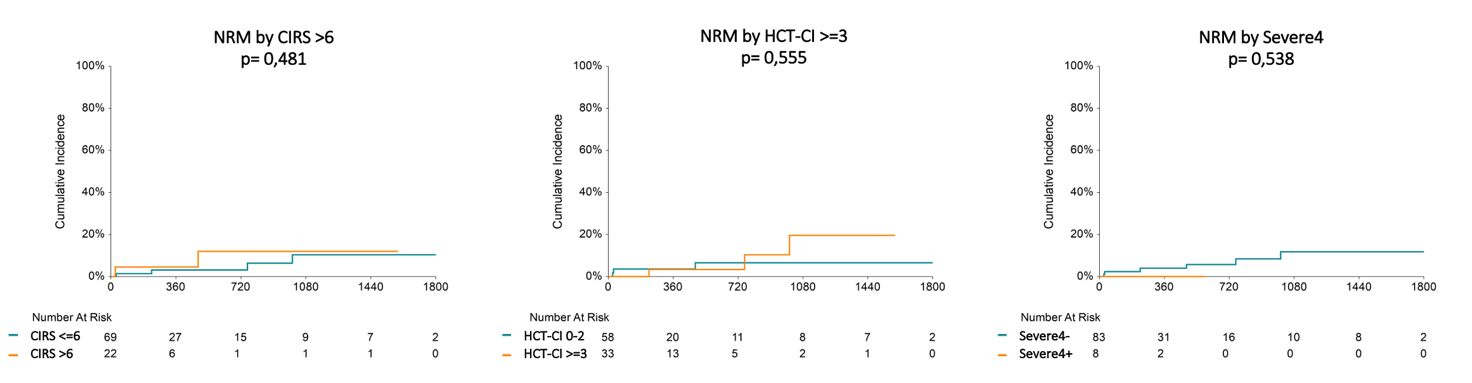
**
